# Spatial and cross-sectoral input spillover effects: the case of the Italian tourism industry

**DOI:** 10.1007/s11123-023-00665-4

**Published:** 2023-02-24

**Authors:** Silvia Emili, Federica Galli

**Affiliations:** Department of Statistical Sciences, University of Bologna, P.tta Teatini 10, 47921 Rimini, Italy

**Keywords:** Productivity, Cross-sectoral spillovers, Input spillovers, Tourism segments, Local units

## Abstract

The aim of this paper is to extend the literature on multisectoral industries productivity such as tourism by simultaneously analysing the multidimensional nature of the Italian tourism sector in the period 2011–2020, considering both cross-sectoral and spatial spillover effects. To further improve our analysis, we consider two fundamental features for policy decisions: high spatial detail of analysis and the multipurpose nature of the tourism industry. Empirical findings confirm the hypotheses that the productivity level of the Italian tourism industry depends on its ability to make to most of the different input factors coming from different sectors and on (positive and negative) input spillovers.

## Introduction

Analysing the productivity of industries and economic systems is a fundamental measurement aspect for the economic wealth of international, national, regional and local territories. At the industrial level, a general definition of productivity relates to the capability of a firm to use and combine input factors for the production of a certain output. This general definition encounters several complications as the reliability of the models and the results become central to the policy decision making processes. The geographic amplitude of the systems, the interactions between competitors, cooperative and public agents, the limitation of resource conditions, and the improvement in production processes and knowledge mechanisms, these are only a limited set of issues that may arise in the investigation of productivity. This improvement in the complexity of empirical investigations accompanies the need for policymakers to understand, develop and sustain the current productivity level of the industries in view of long-term economic growth and per capita income growth.

One of the main relevant (and challenging) sectors in the world economic balance for production analysis applications is clearly the tourism sector. The World Travel and Tourism Council (WTTC) estimated the contribution of the Travel & Tourism sector as 10.4% of global GDP in 2019, and 5.5% in 2020, with a loss of US$ 4.7 trillion in this last year. In terms of job creation, the WTTC said the sector accounted for one in four of all the new jobs created prior to the pandemic. Italy represents a meaningful case study to analyse tourism productivity, in the first place, because of its impact on total GDP. According to the National Agency for Tourism (hereinafter ENIT), in 2019 the Italian tourism industry accounted for 13% of Italy’s GDP, providing 4,2 million employees (ENIT 2020).

The challenge in analysing tourism system productivity can be easily seen starting from the definition of the tourism system itself. The World Tourism Organization (UNWTO) defines tourism industries^1^ as comprising “*all establishments for which the principal activity is a tourism characteristic activity. Tourism industries (also referred to as tourism activities) are the activities that typically produce tourism characteristic products.”*

In this way, it seems natural to relate the investigation of tourism productivity to multi behavioural conceptualisation tools (Buhalis 2000; Haugland et al. 2011; Murphy et al. 2000), such as network analysis (Baggio et al. 2010; Dwyer et al. 2003; Kim et al. 2021), in the perspective of co-production and collaboration of different actors for the total destination product.

The complexity of the network can be described by three main features: (i) multisectoral and multiproduct production processes (Otto and Ritchie 1996), (ii) spatial phenomena such as agglomeration and clustering (Kim et al. 2021; Li and Liu 2021), and (iii) contextual factors and physical characteristics of the destination identifying specific tourism segments (Yang 2016). Despite the relevance of these three aspects, to our knowledge none of the studies in the literature analysing tourism productivity attempted to match and combine all of them.

Considering the first of the three aspects, it is well recognised that tourism is a “*multi-sectorial and inseparable sector*” (Hor 2021, p.1) that integrates various activities such as travel, accommodations, restaurants, art and entertainment. Indeed, it does not appear as a unique sector nor in the input-output tables nor in national account systems (Fletcher 1989; Teigeiro and Diaz 2014). A fundamental profitable opportunity for the actors in this industry can therefore be represented by a wider access to a heterogeneous set of resources, a higher level of flexibility and responsiveness of the system and a greater openness to new technologies and services. The evolutionary aspect of the multi-sectoral connotation of the tourism industry relates to the increased possibility of new working relationships, learning and innovation practices, knowledge transfer (Baggio and Cooper 2010; Fleming and Marx 2006), and to a greater access to promotion and commercialisation campaigns, complementary assets, and co-joint actions (Ndou and Passiante 2005). Then, the longevity and the strength of the structure of the relationships, or in other words, the social capital (Coleman 1988) of the tourism destinations can turn into a massive channel for value creation, leading to the need of investigating and understanding the linkages that support tourism value creation (Du and Zhao 2013).

The second aspect related to the complexity of networks in tourism economic systems is related to spatial phenomena such as agglomeration and clustering (Kim et al. 2021; Li and Liu 2021): along with the identification and investigations of spillover mechanisms and channels between related subjects and the nontrivial investigation of productivity, the awareness that the geographical dimension and the investigation of tourism productive systems is important raises the bar to the development and application of adequate statistical and econometric methods for analysis (Assaf and Dwyer 2013; Joppe and Li 2016; Pham 2020) among which spatial models (Yang and Wong 2012; Kim et al. 2021).

In this work, we improve the level of analysis developed in the literature, considering a dynamic spatial panel data model (Elhorst 2013) where sectoral-specific input spillovers are evaluated together with cross-sectoral linkages. Most research on tourism productivity from a regional viewpoint uses linear regressions considering different functional forms, estimation approaches, and variables of interest. However, in line with Kim et al. (2021), the following analyses will reveal the fundamental role of both temporal and spatial components in deepening the relationships characterising the productive process of a tourism system. The starting point of this study is to support the idea that ignoring the dynamics and spatial dependences could offer misleading estimates, depicting the reliability of the results obtained with the adopted methodology over more standard approaches.

The third aspect describing the complexity of the tourism system, and representing the main challenge of this work, concerns the strong connection of the tourism industry to the contextual factors and the physical characteristics of the destination (Yang 2016). The relevance of this feature can be easily appreciated for Italy. The country is a clear example of tourism product fragmentation and heterogeneity (Sainaghi and Baggio 2017). These two features of the tourism supply are usually found in countries with economic balances and national accounts that are strongly related to the tourism sector (Haugland et al. 2011; Pearce 1989). Here, the national and regional government policies and business strategies are combined with the capacity of local agents and stakeholders to provide, sustain and implement adequate instruments that adapt to the specificities of the destinations (Whitford 2004) as a diversified portfolio of goods and services for a pluralism of customers with different needs and preferences (Romão et al. 2015). Local communities, stakeholders and public agents play a key role in developing attractive tourism experiences around the physical, cultural and geographical characteristics of the territory (Sainaghi and Mauri 2015). The singularities of the local destinations and of the different processes of collaboration and co-production of tourism-related industries, generate several questions on the possible differences in productivity and spillover effects of destinations belonging to different tourism segments (e.g. cultural and heritage, sport, religious tourism).

Thus, motivated by the multiple-purpose nature of the Italian tourism industry, this work aims to provide evidence of substantial differences in spillover mechanisms when differentiating between three important segments of Italian tourism activity: large cities and destinations with a cultural, historical, or artistic offerings (hereinafter identified as *cities*), maritime destinations (*sea*), and lake, thermal, mountains and scenic destinations (*mountain*). In particular, this study does not just look at whether there are spatial interdependencies or dynamic effects in the Italian tourism system as a whole but also investigates the heterogeneity by destination typology.

In sum, using aggregated data on the consolidated accounts of different tourism sectors at the local labour systems level for Italy in the period 2011–2020, we investigate the productivity performance of the tourism industry, differentiating between tourism segments, using a spatial econometric approach. In particular, this is the first contribution modelling cross-sectoral and spatial spillover effects together in the Italian tourism industry, further combining two fundamental features for policy decisions: high spatial detail of analysis and the multipurpose nature of tourism destinations.

Indeed, the choice of Local Market Areas (hereinafter, LMA) as spatial detail of the analysis merged the importance of considering the destination as a fundamental unit in several economic and managerial branches^2^, and the idea of capturing economic and socio-cultural aspects and patterns in the tourism production process that couldn’t be observed at regional or national level (Bernini and Guizzardi 2010; Ma et al. 2015; Yang 2016). Then, even if this spatial detail is not unusual for different purposes in the tourism literature (for a review see Lazzeretti et al. 2008), LMAs have never been considered as spatial statistical units in a tourism productivity analysis.

The results will offer a new perspective for policymakers guiding them in taking advantage of existing spatial diffusion or competition processes occurring across nearby destinations, and to design appropriate cluster-based developing programs in order to support all segments and sectors of the Italian tourism industry both in the short and in the long run.

## Literature

The economic relevance of the spatial and industry inter-dependencies in understanding production processes and performance is, now, widely recognised and takes a central role in different strands of the economic literature such as geographic and regional sciences (Cohen and Morrison Paul 2005; Badinger and Egger 2016) and tourism (Jackson and Murphy 2002; Michael 2003; Sölvell et al. 2008; Yang 2012). Agglomeration economies and clusters are particularly relevant in the tourism sector because the service offered is inseparable in time and space and because tourism demand and supply are localised in specific concentrated places (Majewska 2017).

Tourism destinations can be seen as forms of industrial clusters (Jackson and Murphy 2002; Shaw and Williams 2009), made up of groups of SMEs that cooperate to build up a successful tourism product (Novelli et al. 2006; Jackson and Murphy 2006). Tourism clusters are defined as a set of linked activities such as accommodations, attractions, services, tour operators, travel agents and complementary products that contribute to the tourism experience (Wang and Fesenmaier 2007). Therefore, tourism clusters, through networking, alliances, active collaboration and innovation can succeed in successfully competing in the global tourism market through local cooperation (Smeral 1998) by accumulating new knowledge and innovating more easily than isolated firms (Marco-Lajara et al. 2019).

Investigating the structure of tourism clusters, Michael (2003) identified tourism clusters as diagonal clusters. Differently from horizontal and vertical clusters that refer to the co-location of firms selling the same products and to the co-location of an industry’s supply chain respectively, diagonal clusters are characterised by the concentration of complementary or symbiotic firms. Therefore, even if the products offered can be quite different, each firm adds value to the activity of the other firms, creating a network in which separate products and services are linked together to form a unique item. In this framework, a tourism destination can be seen as a diagonal cluster in the sense that different industries belonging to the same destination work together to build up a valuable tourism experience.

In addition to the cross-sectoral feature of tourism clusters, a consistent number of works identified the key role of spatial proximity occurring among neighbours in assessing agglomeration and concentration effects in tourism destinations (Zhang et al. 2015; Marco-Lajara et al. 2016, 2019). Tourism clusters, likewise manufacturing clusters, benefit from the existence of positive spatial feedbacks resulting from proximity, trust, and common values that boost collaboration, social contact, and emulation among neighbours (Shaw and Williams 2009). As a consequence, clustered tourism firms experiment better performances thanks to improved knowledge, shared ideas and innovations (Adam and Mensah 2013) and spontaneous concentration processes generating from positive customers feedbacks in terms of demand (Yang 2012).

Therefore, the combination of services and products emerging from the cooperation between firms located in neighbouring areas can be seen as the result of both sectoral linkages and spatial proximity relationships (Kim et al. 2021). Haugland et al. (2011, p. 269) stress the idea that “*The success of individual actors, as well as the success of the entire destination, is dependent on efficient coordination and integration of individual companies’ resources, products, and services*”. The two kinds of relationships are naturally generated and reinforced over the years by phenomena such as sharing facilities and infrastructure (Bramwell 2004), coordination in promotional programmes for local, historical, geographical and physical appeal of neighbouring destinations (Yang 2016), and commonalities in production and knowledge processes that may affect the performance of a specific destination with potential contributions from neighbouring economies via tangible and intangible interactions (Shaw and Williams 2009).

To investigate sectoral linkages and spatial proximity relationships as sources of production spillover effects, different advanced methodological tools have been considered by scholars. Classical instruments for linkage investigations are input-output analysis (Teigeiro and Díaz 2014; Yan and Wall 2002), Tourism Satellite Account (Frechtling 2010; Figini and Patuelli 2021; Smeral 2006), and computable general equilibrium (Dwyer et al. 2004; Dwyer 2015; Van Truong and Shimizu 2017). On the other hand, the literature analysing the impact of spatial relationships on tourism productivity is still developing (see Yang and Wong 2012; Kim et al. 2021).

Very recently, Kim et al. (2021) considers a spatial panel data model to account for spatial spillovers in tourism productivity analysis. The authors illustrate a need to estimate the impact of agglomeration phenomena on production performance, explicitly accounting for a spatial dependency structure. Using data on local authority districts (LAD) of the UK for the period 2006–2016, Kim et al. (2021) show the role of spatial spillovers on labour productivity estimating a dynamic spatial panel model to “*capture the dynamic structure of agglomeration and its effects on labour productivity of tourism firms*”. Their findings lend general support to agglomeration literature, in view of a statistically significant impact of direct and indirect effects on production within a LAD and across neighbouring LADs. However, the analysis completely neglects the multisectoral nature of the industry, focusing exclusively on the aggregate spatial effects.

Therefore, in this work, we extend previous literature in this field by evaluating both cross-sectoral and spatial linkages affecting the overall output of the Italian tourism industry.

In particular, we first empirically verify that the performance of a certain destination is determined by (i) the ability of tourism systems to make the most of input factors and by the level of production gained the previous year, but also by (ii) the input spillovers from neighbouring destinations. Then, we formulate the following research hypotheses:

H1: Destinations belonging to different tourism segments exploit the input factors differently.

H2: There are substantial differences in spatial spillover effects across different tourism segments.

In particular, our analysis allows identifying input spillovers occurring across neighbouring destinations and the specific linkages in term of value creation characterising the different sectors in the tourism industry. To reach this goal, we estimate a Cobb-Douglas production function using a dynamic spatial approach and we take advantage of georeferenced balance sheets data on Italian tourism firms at LMA level in the time period 2011–2020.

## The model

To model productivity accounting for both spatial spillovers and cross-sectoral relationships, the first step is choosing a flexible but straightforward production function. Therefore, a model specification based on the Cobb-Douglas family was selected where the output of each tourism destination as a multiplicative function of input factors is defined as coming from the main sectors involved in the final total production. The estimated form of the Cobb-Douglas function, obtained after the logarithm transformation, for each unit *i* = 1, …, *N* observed at time *t* = 1, …, *T* belonging to the sector *s* = 1, …, *S*, is defined by:

1 $$\begin{array}{*{20}{c}} {\ln Q_{it} = \beta _0 + \mathop {\sum}\limits_s {\left( {\beta _1\ln K_{its} + \beta _2\ln L_{its}} \right) + {{\epsilon }}_{it},} } & {{{\epsilon }}_{it} \sim iid\left( {0,\sigma _{{\epsilon }}^2} \right)} \end{array}$$

where *Q*_*it*_ is the output of the *i*th LMA modelled as function of two inputs, capital *K*_*its*_ and labour force *L*_*its*_, widely identified by the literature as key factors in the production process of tourism-related firms (see Bernini and Guizzardi (2010) and references therein). The reason why we consider the output of the production function in Eq. (1) as the total output at the destination level instead of using the sectorial one is twofold: first, the model aims to provide a unified tool useful for policymakers to develop (multi-) destinations-oriented programs able to both engage in cooperative destination marketing actions and bolster the overall tourism destination competitiveness; second, from an econometric point of view, the analysis of production functions separated by sectors may be unreliable due to the omission of possible interactions between sectorial outputs. This kind of analysis would require the investigation of possible simultaneous effects and then the adoption of restrictive assumptions. Even if an appropriate solution would concern the application of a system of dynamic spatial panel data model (Elhorst and Emili 2022) the limitation in data availability over time, may cause inconsistent and biased estimates.

While the multisectoral nature of tourism is captured by distinguishing the inputs aligned with the sectors in tourism production, the second aspect, i.e. the spatial dimension, is captured by expanding Eq. (1) to model spatial phenomena. The specific class of spatial econometric approaches (Elhorst 2013) considered in this study is the dynamic spatial Durbin models (dynamic SDM). The general idea of the SDM specification is to model the dependent variable observed for a specific region *r* as a function of a set of covariates for both the region *r* and its neighbours, and of the dependent variable of neighbouring units. Therefore, the estimated Cobb-Douglas function takes the form:

2 $$\begin{array}{l}lnQ_{it} = \alpha lnQ_{it - 1} + \rho WlnQ_{it} \\ \qquad\quad\,\, + \mathop {\sum}\limits_s \left(\right. lnK_{its}\beta _{1s} + lnL_{its}\beta _{2s} + WlnK_{its}\delta _{1s}\\ \qquad \quad\, \, +\, WlnL_{its}\delta _{2s} \left)\right. + \,u_i + \eta _t + \nu _{it}, \quad {\nu _{it} \sim iid\left( {0,\sigma _\nu ^2} \right)} \end{array}$$

where the logarithm of the output, i.e. *lnQ*_*it*_ is proxied by the aggregated total value added for the LMA *i* (defined as the sum of the outputs of each sector) and the input factors entering the tourism production process are given by the logarithmic transformations of the number of employees (*lnL*_*its*_) and of the amount of fixed assets (*lnK*_*its*_) aggregated by sectors. Fixed assets include all tangible items that a firm purchases and uses in the production process to create its final goods and services (e.g. the building itself, equipment, furniture, etc.). These two variables have been chosen as proxies of labour and capital respectively, in line with previous literature on tourism productivity analysis (Smeral 2007; Roget and Rodriguez-Gonzales 2006). The spatial (deterministic) structure in the data is captured by the spatial weighting matrix W, collecting information on the spatial distribution of LMAs over the Italian territory.

The specification in Eq. (2) allows us to consider the output of the spatial unit *i* at time *t* to be affected by its output observed in the previous time period (*lnQ*_*it*-1_), but also by neighbouring units’ output (*WlnQ*_*it*_), as result of tourism agglomeration effects. In addition, Eq. (2) allows us to model the production of each LMA depending on both its own input factors (*lnK*_*its*_ and *lnL*_*its*_), and the inputs available in neighbouring territories (*WlnK*_*its*_ and W*lnL*_*its*_). The model specification is then completed by introducing the terms *u*_*i*_ and *η*_*t*_ to collect individual and time fixed effects respectively.

An important feature of spatial models is clearly the definition of the spatial structure given by W. The most common spatial weight matrices are binary contiguity of the first or second order and inverse distance matrices. Next to the choice of these “pure” physical weighting matrices, the regional sciences literature is becoming more and more conscious about the relevance of economic-based Ws. Corrado and Fingleton (2012) reviewed the specifications proposed in the last 40 years of research, paying particular attention to the need for spatial weights to account for the possible origins of spillovers such as “*migration, displaced demand and supply effects in the housing market, input–output linkages, competition and coordination between firms, localised information flows through social networks, strategic interaction between policy makers, tax competition between local authorities, or even simply arbitrary boundaries*” (Corrado and Fingleton 2012 p. 216). However, in this case, aware of the complexity of handling different subsectors, different tourism segments, the heterogeneity of the agents involved in the production process and the heterogeneity of the phenomenon across the Italian territory, for parsimony, the choice of a spatial structure based on physical distances should be preferred. In particular, for this analysis the spatial structure is given by a second-order contiguity matrix, aiming to consider relationships between both neighbouring LMAs and neighbours of neighbours. This choice primarily relates to the geographic characteristics of the units. Specifically, as will be amply described in the next sections, the spatial units considered in this study are different in terms of surface area, population, physical connectivity and infrastructures, geographic location and conformity. In this way, the choice for a spatial contiguity matrix instead of a solution built on the physical distance between centroids or main city centres appears to be a more adequate and reliable tool. For the same reason, in certain cases where the territories are closely related to each other in terms of vicinity, accessibility and combination of tourism products and services, the assumption of one unique order of contiguity can be too restrictive. Nevertheless, in the following sections, we also provide insights on the robustness of the final solution considering a first-order contiguity W.

## Data

Following Lazzeretti and Capone (2008) for the measurement of tourism supply in terms of employees per sector, in this study, we use Italian data collected at individual levels for different sectors of the tourism industry, and then we aggregate them according to LMAs, typical of district analyses. The aggregation allows us to focus on the agglomerative effects of the destination economies that create and then suffer the input spillover effects from neighbouring areas. Specifically, the data used for the analysis are collected from the Aida-Bureau Van Dijk database, which is the only available dataset containing georeferenced data on the consolidated accounts of Italian companies. To proxy the entire tourism industry, we concentrated on tourism firms belonging to the five main sectors contributing to the whole Italian tourism offer (Figini and Patuelli 2021). Table A1 in Appendix shows the list of the tourism products reported in the Tourism Satellite Accounts according to Eurostat (2014). From this list, we excluded the travel agency sector since it relates to the outbound tourism and is not associated with the LMA tourism production. Therefore, following the Italian system nomenclature of economic productive activities ATECO, we consider the accommodation sector (ATECO 55, hereinafter A*ccommodation*), the restaurant sector (ATECO 56, *Restaurant*), the creative, arts, and entertainment sector (ATECO 90, C*reative&Arts*), the recreation and entertainment sector (ATECO 93, *Entertainment*), and the transport sector (ATECO 49-50-51, *Transport*). *Entertainment* includes free time activities such as gyms, sports facilities, sports clubs, bathing facilities, game rooms, discos, and night clubs while the *Creative&Arts* generally covers cultural activities such as artistic and literary representations, live performances, and events for the public. Finally, in *Transport* we only considered passenger rail and air transport, and sea and coastal passenger transport, excluding freight transportation firms.

As described earlier, we implemented our analysis focusing on the Italian labour market areas (LMAs) identified by the Italian National Institute of Statistics (ISTAT). According to the most recent information available from 2018, the LMAs are defined as 610 sub-regional geographical units in which most of the labour force lives and works, and where companies can find the largest amount of the labour force necessary to fulfil the offered jobs. In particular, the LMAs have been defined to meaningfully compare different sub-regional labour market areas without necessarily respecting the administrative boundaries. Thus, they can be considered the best and finest aggregate level to identify economies that are effective in terms of type and scope of tourism destinations. Hence, by aggregating the AIDA georeferenced firm-level microdata by sector and spatial unit, we obtained a balanced panel of 607 LMAs^3^ covering the period 2011–2020 for each ATECO sector considered in the analysis.

### Descriptive statistics

**Fig. 1:**
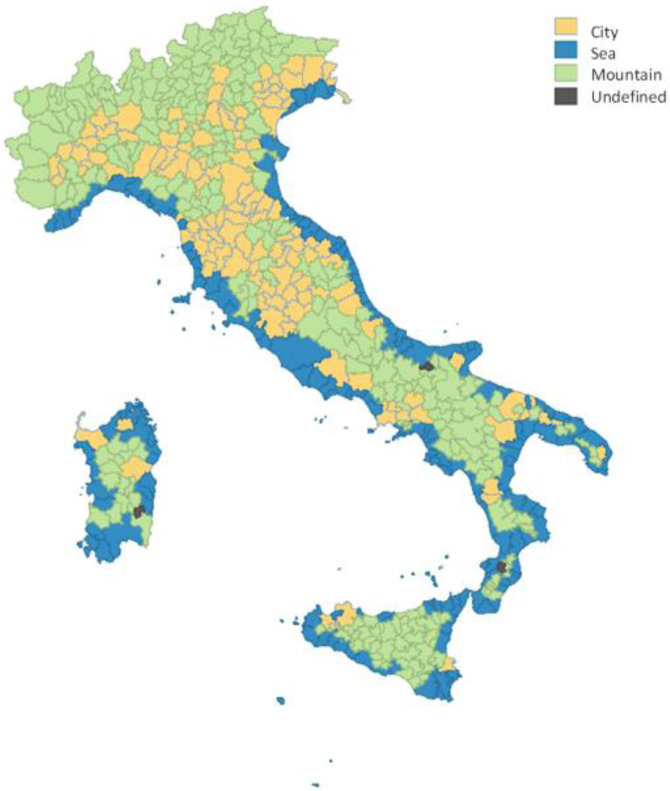
Classification of tourism destinations

In this paragraph, we summarise the variables used to analyse the relationship between the overall tourism sector output and the inputs (i.e. labour and capital) of the five different sectors considered. For each LMA we defined the overall tourism sector value added as the sum of the value added of the *Accommodation*, *Restaurant*, *Creative&Arts*, *Entertainment* and *Transport* industries, differentiating the LMAs across three main tourism destination typologies: big cities and destinations with cultural, historical, or artistic offerings (for brevity, *cities*), maritime destinations (*sea*), and lake, thermal, mountain and scenic destinations (*mountain*). Our classification of the different LMAs by destination typology is based on the 2019 ISTAT tourism municipality classification. The Institute classifies each municipality according to the prevailing tourism segment in the territory. Thus, to aggregate this information at LMA level we assigned to each spatial unit the prevailing destination typology in the area using the municipal surfaces as weights. The resulting classification is shown in Fig. 1.

Without distinguishing among the segments, we see in Table 1 that the *Transport* and *Restaurant* sectors mostly contribute to generating the overall added value in the tourism industry, followed by *Accommodation*, *Entertainment*, and *Creative&Arts*. Focusing on the different tourism destination typologies, *city* destinations are on average the most profitable for the Italian tourism sector in terms of value added, followed by *sea* and *mountain* destinations. Moreover, for the *city* segment, the *Restaurant* sector is the prevailing one in terms of generated value added followed by *Transports*, while for the *sea* and *mountain* destinations the *Transport* industry is the most substantial along with *Accommodation*. The *Restaurant* sector provides employment to the greatest number of people in the tourism industry, while *Transport* and *Accommodation* account for the majority of investments in fixed capital. In particular, in *city* and *mountain* destinations the *Accommodation* sector doubles *Transport*, yet for *sea* destinations investments in fixed capital in *Transport* exceed those of the *Accommodation* sector.

**Table 1:** Descriptive statistics

	Overall		City		Sea		Mountain	
	Mean	SD	Mean	SD	Mean	SD	Mean	SD
Q (Overall)	41,435	274,453	72,861	304,685	57,128	411,309	16,506	65,685
Q_Accommodation_	8570	38,181	13,548	61,495	11,497	40,951	4330	14,119
Q_Restaurant_	11,039	78,165	29,640	148,471	8927	58,846	3892	15,231
Q_Creative&Arts_	1025	8308	3101	16,312	720	5100	261	1761
Q_Entertainment_	4983	31,764	9055	39,439	5739	34,301	2592	25,134
Q_Transport_	15,764	160,950	17,517	58,942	30,245	282,573	5430	25,529
L_Accommodation_	213	812	312	1208	280	918	113	317
L_Restaurant_	530	3354	1362	6261	448	2632	189	656
L_Creative&Arts_	58	288	122	468	44	206	21	74
L_Entertainment_	80	293	148	411	92	342	36	126
L_Transport_	301	2113	427	1212	433	3425	130	495
K_Accommodation_	47,638	201,616	74,071	280,414	63,170	250,860	23,011	64,759
K_Restaurant_	8609	39,442	18,035	68,893	8087	36,543	4471	11,262
K_Creative&Arts_	1471	7000	2835	10,303	1014	4500	789	5122
K_Entertainment_	6784	28,662	11,842	35,017	7334	36,599	3761	14,539
K_Transport_	51,729	584,246	30,804	135,635	114,524	990,487	13,098	39,939

*Y* value added in thousands of euros, *L* number of employees, *K* fixed capital in thousands of euros

Lastly, Table 2 shows the dynamics of the variations (in percentage) in the value added over the years 2012–2020. Only 2012 shows a negative variation (−0.60%), due to the crisis of the sovereign debt and 2020 due to the global Covid-19 pandemic (−52.08%). The Covid-19 pandemic cut the tourism sector’s value added in half for 2020 compared to 2019 due to a sharp fall in profitability across all five ATECO sectors considered, with the highest reductions in *Accommodation* (−66.35%), *Restaurants* (−54.23%), and *Transports* (−48.88%). These preliminary analyses are then completed by providing a correlation matrix between the outputs and the inputs of the different sectors and some scatterplots, respectively shown in Table A2 and Fig. A1 of the Appendix. Both instruments suggest a strong association between the outputs and the inputs, finding support for the following investigations.

**Table 2:** Percentage variation over years

	2012	2013	2014	2015	2016	2017	2018	2019	2020
Q (Overall)	−0.60	3.53	5.19	10.72	6.05	9.29	4.63	6.85	−52.08
Q_Accommodation_	−0.71	5.75	4.96	14.97	10.67	8.35	4.80	4.40	−66.35
Q_Restaurant_	−1.25	10.63	1.68	6.35	17.21	14.54	5.20	12.42	−54.23
Q_Creative&Arts_	−8.65	10.25	1.84	10.84	8.89	5.20	−2.85	26.30	−42.36
Q_Entertainment_	1.24	−0.17	12.40	8.49	11.27	15.47	1.33	8.30	−32.98
Q_Transport_	−0.15	−1.04	5.86	11.93	−4.54	4.33	5.76	2.24	−48.88

### Preliminary spatial analysis

**Fig. 2:**
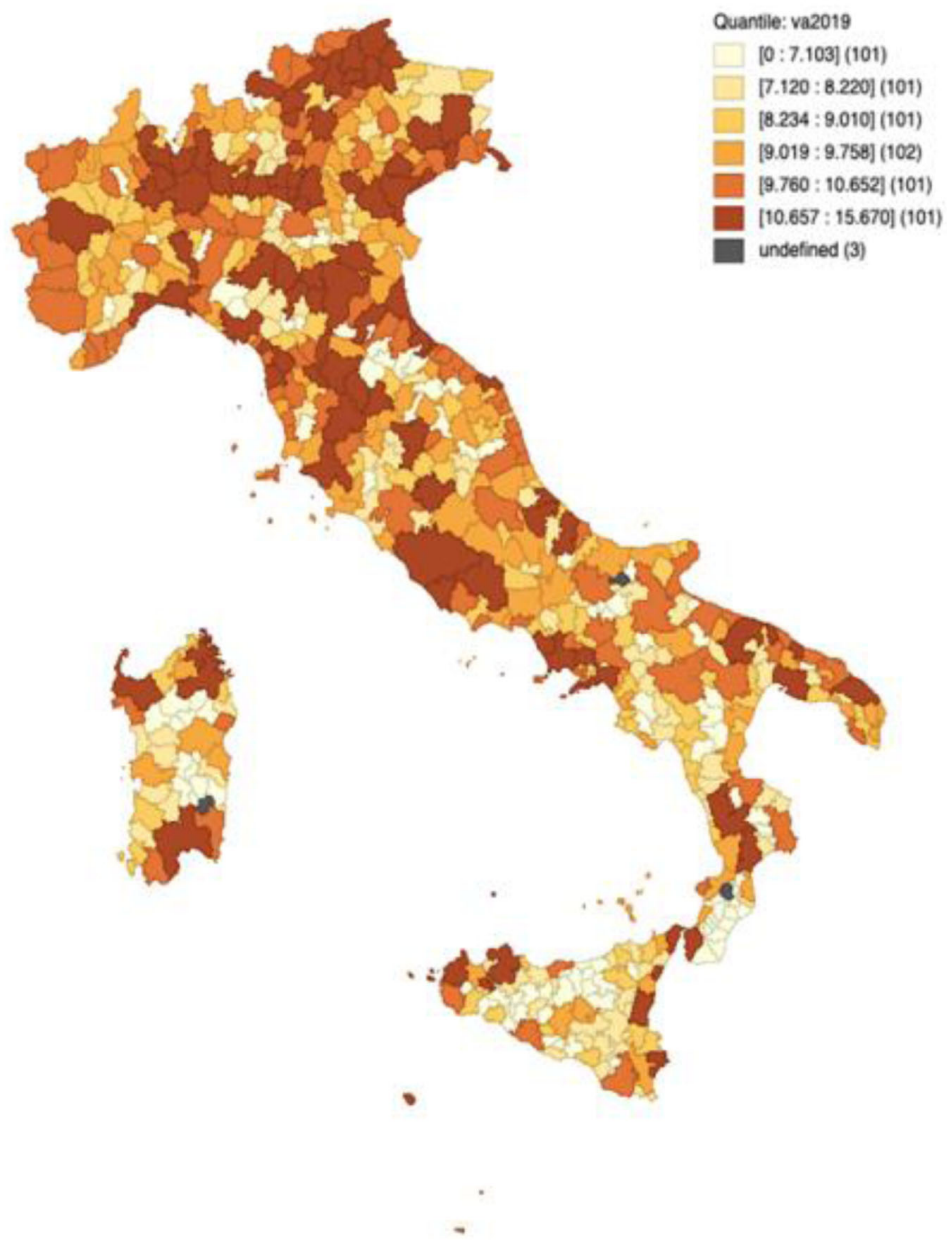
Quantile map: value added 2019

Figure 2 shows the tourism sector value added quantile map for the year 2019. See Fig. A2 in the Appendix for the regional divisions in Italy. Overall, the North and the Centre are the most profitable macro areas of Italy. Specifically, the most productive areas are found in the regions of Trentino Alto Adige, Liguria, Emilia Romagna and Tuscany as well as the coastal areas of Veneto and Friuli Venezia Giulia, and the LMAs in the neighbourhoods of Milan, Rome, and Naples. Focusing on the South of Italy, only the southern part of Apulia, and some coastal LMAs in Sicily and Sardinia are as productive as the northern destinations. It is noticeable that the most productive spatial units tend to concentrate as do the less productive LMAs. For example, all the LMAs inside Sicily and Sardinia, the heel of Calabria, and the Apennine territory tend to reach very low levels of value added.

Further support to the existing global and local spatial correlation between neighbouring LMAs can be found considering (respectively) the results of the Moran test, reported in Tables A3 and A4 in Appendix and the LISA significance cluster map (Anselin 1996) shown in Fig. A3 in Appendix. At the global level, the Moran test calculated both on the output and input variables allow us to reject the null hypothesis of spatial randomisation (absence of autocorrelation) in favour of the presence of spatial linkages in the data. Concerning possible differences in the spatial structure at the local level, the LISA maps for both the overall sample and the specific tourism segments show that both when considering the overall tourism sector and the three destination typologies separately, the nearest LMAs are affected by significant local spatial dependence confirming previous insights from Fig. 2.

## Results

Tables 3 and 4 show the estimation results of the dynamic SDM model for the overall tourism industry and the three different tourism segments (i.e. *city*, *sea*, and *mountain*) respectively. Table 3 also includes the estimates of several nested specifications such as the dynamic and static spatial autoregressive model (SAR), the dynamic and static SLX, and dynamic and static specification without spatial components (OLS), as well as some goodness of fit statistics. For further details on model specifications of spatial panel data models see Elhorst (2013).

**Table 3:** Estimates of spatial models

	Dynamic SDM	Dynamic SAR	Dynamic SLX	Static SDM	Static SAR	Static SLX	Dynamic No spat.	Static No spat. (OLS)
N. obs = 6070								
lnQ_t-1_	0.47*** (0.01)	0.41*** (0.01)	0.30*** (0.04)	–	–	–	0.47*** (0.03)	–
WlnQ_t_	0.37*** (0.03)	0.42*** (0.02)	–	0.25*** (0.01)	0.34*** (0.02)	–	–	–
lnL_Accommodation_	0.10*** (0.01)	0.10*** (0.01)	0.05** (0.02)	0.14*** (0.01)	0.15*** (0.01)	0.19*** (0.01)	0.04** (0.02)	0.21*** (0.01)
lnL_Restaurant_	0.10*** (0.02)	0.07*** (0.02)	0.14*** (0.03)	0.14*** (0.02)	0.16*** (0.01)	0.19*** (0.02)	0.20*** (0.02)	0.23*** (0.01)
lnL_Creative&Arts_	0.01 (0.01)	0.01 (0.01)	0.02** (0.01)	0.01 (0.01)	0.01 (0.01)	0.02* (0.01)	0.02*** (0.01)	0.02** (0.01)
lnL_Entertainment_	0.02* (0.01)	0.01 (0.01)	0.02* (0.01)	0.03** (0.01)	0.03*** (0.01)	0.05*** (0.01)	0.01 (0.01)	0.06*** (0.01)
lnL_Transport_	0.11*** (0.01)	0.12*** (0.01)	0.08*** (0.02)	0.14*** (0.01)	0.14*** (0.01)	0.16*** (0.01)	0.10*** (0.02)	0.16*** (0.01)
lnK_Accommodation_	0.07*** (0.01)	0.09*** (0.01)	0.06*** (0.01)	0.09*** (0.01)	0.10*** (0.01)	0.11*** (0.01)	0.04*** (0.01)	0.11*** (0.01)
lnK_Restaurant_	0.11*** (0.01)	0.13*** (0.01)	0.04** (0.02)	0.15*** (0.01)	0.15*** (0.01)	0.15*** (0.01)	0.05*** (0.02)	0.15*** (0.01)
lnK_Creative&Arts_	0.01 (0.01)	0.01 (0.01)	0.00 (0.01)	0.02** (0.01)	0.02** (0.01)	0.01 (0.01)	0.00 (0.01)	0.01 (0.01)
lnK_Entertainment_	0.03*** (0.01)	0.04*** (0.01)	0.03*** (0.01)	0.04*** (0.01)	0.04*** (0.01)	0.05*** (0.01)	0.02** (0.01)	0.05*** (0.01)
lnK_Transport_	0.01** (0.01)	0.02*** (0.01)	0.04*** (0.01)	0.02*** (0.01)	0.02*** (0.01)	0.02*** (0.01)	0.04*** (0.01)	0.03*** (0.01)
WlnL_Accommodation_	0.04 (0.05)	–	0.08* (0.04)	0.01 (0.05)	–	0.10** (0.04)	–	–
WlnL_Restaurant_	−0.32*** (0.04)	–	0.07* (0.04)	0.10** (0.04)	–	0.09** (0.04)	–	–
WlnL_Creative&Arts_	0.00 (0.04)	–	−0.03 (0.03)	−0.02 (0.04)	–	−0.03 (0.04)	–	–
WlnL_Entertainment_	0.01 (0.04)	–	−0.09*** (0.03)	−0.03 (0.04)	–	−0.04 (0.04)	–	–
WlnL_Transport_	0.22*** (0.05)	–	0.12** (0.05)	0.08 (0.05)	–	0.11** (0.04)	–	–
WlnK_Accommodation_	0.15*** (0.02)	–	0.04 (0.03)	0.04* (0.02)	–	0.03 (0.02)	–	–
WlnK_Restaurant_	0.07** (0.03)	–	0.06 (0.04)	−0.01 (0.03)	–	0.01 (0.03)	–	–
WlnK_Creative&Arts_	0.04 (0.03)	–	0.01 (0.03)	0.06** (0.02)	–	0.01 (0.02)	–	–
WlnK_Entertainment_	0.09*** (0.02)	–	0.08*** (0.02)	0.03 (0.02)	–	0.01 (0.02)	–	–
WlnK_Transport_	−0.07** (0.03)	–	0.01 (0.02)	−0.04 (0.03)	–	−0.01 (0.02)	–	–
LL	−3013.64	−3060.42	−4475.55	−3846.78	−3862.34	−5135.83	−4555.71	−5216.19
LR	–	93.56***	2923.82***	1666.28***	1697.40***	4244.38***	3084.14***	4405.10***
AIC	6071.28	6144.84	8993.10	7735.56	7746.68	10311.66	9133.42	10519.49
BIC	6218.92	6225.37	9134.03	7876.49	8820.50	10445.88	9207.24	10519.49

Standard errors in brackets

*p < 0.10; **p < 0.05; ***p < 0.01

**Table 4:** Estimates of the dynamic SDM for the three tourism segments

	City		Sea		Mountain	
	N. obs = 1330		N. obs = 1880		N. obs = 2860	
	Coeff.	SD	Coeff.	SD	Coeff.	SD
lnQ_t-1_	0.20***	0.03	0.28***	0.03	0.47***	0.02
WlnQ_t_	0.27***	0.04	0.19***	0.03	0.18***	0.03
lnL_Accommodation_	0.07***	0.02	0.25***	0.02	0.10***	0.02
lnL_Restaurant_	0.18***	0.02	0.12***	0.02	0.13***	0.03
lnL_Creative&Arts_	0.01	0.01	0.02	0.02	0.02	0.02
lnL_Entertainment_	0.08***	0.01	−0.00	0.02	0.02	0.02
lnL_Transport_	0.15***	0.02	0.14***	0.02	0.10***	0.03
lnK_Accommodation_	0.04***	0.01	0.08***	0.01	0.08***	0.01
lnK_Restaurant_	0.10***	0.02	0.05***	0.01	0.14***	0.01
lnK_Creative&Arts_	0.01	0.01	0.00	0.01	0.01	0.01
lnK_Entertainment_	0.02	0.01	0.05***	0.01	0.05***	0.01
lnK_Transport_	0.04***	0.01	0.01	0.01	0.03**	0.01
WlnL_Accommodation_	0.10**	0.03	0.01	0.04	−0.00	0.05
WlnL_Restaurant_	−0.13***	0.04	−0.10***	0.04	−0.11*	0.06
WlnL_Creative&Arts_	0.02	0.03	0.02	0.03	−0.07	0.06
WlnL_Entertainment_	−0.01	0.03	−0.03	0.03	0.06	0.04
WlnL_Transport_	0.13***	0.04	0.04	0.03	0.03	0.06
WlnK_Accommodation_	−0.11***	0.03	0.08***	0.02	0.08***	0.03
WlnK_Restaurant_	−0.00	0.04	0.08***	0.03	0.11***	0.03
WlnK_Creative&Arts_	0.02	0.02	0.04**	0.02	0.08**	0.03
WlnK_Entertainment_	0.04*	0.02	0.06***	0.02	0.10***	0.03
WlnK_Transport_	0.01	0.03	−0.04**	0.02	−0.01	0.03

*p < 0.10; **p < 0.05; ***p < 0.01

The first step in the analysis of the estimation outputs of Table 3 concerns the comparison of the results obtained via the standard least square estimation (last column of the table) with the more advanced dynamic spatial model estimates. In general, the absence of both the temporal and spatial lag leads to overestimated coefficients and thus, possible misleading results. Second, we check for possible multicollinearity issues using the variance inflation factors (VIF). Considering a standard level of 10, the results of the VIFs calculated after the least square estimation and reported in Table A5 of the Appendix allow us to refuse the presence of strong multicollinearity between the explanatory variables.

Comparing all the estimated nested models using likelihood ratio tests and the AIC and BIC information criteria, we can conclude that the most comprehensive specification, i.e. the dynamic SDM model (first column of Table 3) is the preferred one. To provide a complete portrait of the phenomenon described by the “best” model, a map of the estimated individual fixed effects is shown in Fig. A4 in the Appendix. According to the figure, the value added of the tourism industry tends to be lower in internal LMAs and in local areas located on the Tyrrhenian coast. On the other hand, LMAs containing bigger cities such as Rome, Palermo, Milan and Turin and those located on the Adriatic coast tend to achieve higher outcome levels.

Starting from the overall tourism sector and referring to the preferred spatial Durbin model, the parameter associated with the spatial lag of the dependent variable (i.e. *WlnQ*_*t*_ in Table 3) reports a positive and significant coefficient in all the estimated nested models, indicating that global spatial productivity spillovers occur in the Italian tourism sector, in line with previous findings on positive agglomeration externalities in tourism (Marco-Lajara et al. 2019). Moreover, for the dynamic models, the parameter related to the time lag of the dependent variable (*lnQ*_*t*-1_) always shows a positive and significant coefficient meaning that the value added at a given year depends highly on past values. Regarding the different tourism segments, despite the global spatial dependence always resulting positive and significant as in the overall model, the parameter for the spatial lag of *lnQ*_*t*_ reaches higher values for city and cultural destinations (0.27) than in maritime and mountain LMAs (0.19 and 0.18 respectively). On the other hand, the coefficient associated with the lagged value of the output appears to be higher in the *mountain* segment (0.47) compared to *city* (0.20) and *sea* (0.28). The idea of evaluating separately the different tourism segments is confirmed by a set of Chow-type tests shown in Table A6 of the Appendix: the appropriateness of our approach is validated since, in most of the cases, the results indicate that the coefficient estimates across types of tourism are statistically different.

However, as strongly asserted in the spatial econometric literature (see LeSage and Pace (2009) for further details), differently from the coefficients obtained through least squares, the estimates in Table 3 related to spatial models including the spatial lag of the dependent variable and in Table 4 cannot be interpreted as marginal effects of the independent variables on *lnQ*_*t*_. Thus, to measure the impact of each element in Eq. (2), and then to interpret the capability of destinations to make the most of different input factors in view of agglomerative economies, it is necessary to compute marginal effects as shown in the next paragraph.

### Marginal effects

To investigate cross-sectoral effects and input spillovers, Table 5 shows the direct and indirect short run and long run effects for the overall model and for the different tourism segments. Differentiating among short run and long run effects, it can be noticed that only the magnitudes of the estimated effects tend to increase with a longer time horizon but neither the sign nor the significance level changes, indicating that both direct and indirect effects become stronger but do not change over time. Moreover, we find that the long run increase in the indirect effects is remarkably higher for the overall sample than for the three single tourism segments due to existing product complementarities and spatial interactions among them. For instance, the indirect effect related to labour input of the transport sector passes from 0.40 to 1.96 overall while it moves from 0.22 to 0.31 in *city*, from 0.07 to 0.13 in *sea*, and from 0.06 to 0.19 in *mountain* destinations. Similar differences are detected also for the negative indirect effect of labour in *Restaurant* and for capital investments in the accommodation sector. Thus, aiming to reach long lasting results, policy makers should implement strategies related to networking and collaboration between neighbouring agents keeping in mind the cumulative effects of spatial spillovers over time. A combination of appropriate strategies focused on both the short term and the long term is therefore of primary interest to local governments. However, to reach greater results in the long-term, collaboration among different tourism segments is required aiming to develop a multi-product tourism offer by strengthening new or existing linkages between neighbouring destinations characterised by different tourism purposes.

**Table 5:** Marginal effects

	Overall		City		Sea		Mountain	
	Direct	Indirect	Direct	Indirect	Direct	Indirect	Direct	Indirect
Short Run								
lnL_Accommodation_	0.10***	0.12	0.07***	0.14***	0.25***	0.07	0.10***	0.02
lnL_Restaurant_	0.10***	−0.45***	0.18***	−0.11**	0.12***	−0.09**	0.13***	−0.10*
lnL_Creative&Arts_	0.01	0.01	0.01	0.03	0.02	0.03	0.01	0.07
lnL_Entertainment_	0.02*	0.02	0.08***	0.02	−0.00	−0.04	0.03	0.07
lnL_Transport_	0.12***	0.40***	0.17***	0.22***	0.15***	0.07***	0.10***	0.06
lnK_Accommodation_	0.08***	0.27***	0.04***	−0.11***	0.09***	0.12***	0.09***	0.11***
lnK_Restaurant_	0.11***	0.17***	0.10***	0.03	0.06***	0.10***	0.14***	0.16***
lnK_Creative&Arts_	0.01	0.07	0.01	0.03	0.01	0.05**	0.01	0.09**
lnK_Entertainment_	0.04***	0.16***	0.02	0.05*	0.05***	0.08***	0.06***	0.12***
lnK_Transport_	0.01	−0.10**	0.04***	0.02	0.01	−0.05**	0.03**	−0.01
Long Run								
lnL_Accommodation_	0.21***	0.71**	0.09***	0.20***	0.35***	0.13**	0.10***	0.09
lnL_Restaurant_	0.15***	−1.66***	0.23***	−0.12*	0.16***	−0.12**	0.25***	−0.18
lnL_Creative&Arts_	0.03	0.07	0.01	0.04	0.03	0.05	0.02	0.17
lnL_Entertainment_	0.04*	0.13	0.10***	0.04	−0.01	−0.05	0.05	0.18
lnL_Transport_	0.27***	1.96***	0.21***	0.31***	0.20***	0.13**	0.20***	0.19
lnK_Accommodation_	0.18***	1.30***	0.05***	−0.15***	0.13***	0.18***	0.17***	0.30***
lnK_Restaurant_	0.23***	0.95***	0.12***	0.05	0.08***	0.16***	0.29***	0.44***
lnK_Creative&Arts_	0.02	0.28	0.01	0.04	0.01	0.07**	0.03	0.21**
lnK_Entertainment_	0.09***	0.75***	0.02*	0.07**	0.07***	0.13***	0.11***	0.32***
lnK_Transport_	0.02	−0.37*	0.05***	0.03	0.01	−0.07**	0.06**	−0.00

*p < 0.10; **p < 0.05; ***p < 0.01

Referring to the hypotheses formulated above, in the next paragraph we first discuss the results related to direct effects aiming to test H1, and then we concentrate on indirect effects, replying to H2.

Starting from the direct effects, as expected, the labour and capital related to the five sectors positively affect the overall performance of both the whole tourism industry and the different segments. Indeed, in line with the concept of diagonal clusters introduced by Michael (2003), a tourism destination can be seen as a composite product in the sense that different industries belonging to the same destination (such as transport, accommodation, restaurants, entertainment and attractions, etc.) work together to reach a greater exposure and to build up a successful tourism experience (Jackson and Murphy 2006). Accordingly, each sector adds value to the activity of the others, creating a network in which separate products and services are linked together to form a unique item. The *Creative&Arts* sector is the only exception, as the direct effect of the workforce and capital endowment shows a non-existent contribution to the economic performance of the destination (i.e. not significantly different from zero) for the input factors of activities such as live production services, theatre events and creative entertainment. Thus, we mostly confirm our first research hypothesis, providing support to the idea that the output of the Italian tourism industry depends on a set of different input factors coming from different sectors, excluding inputs from the *Creative&Arts* industry.

Focusing on the most influential input factors in terms of magnitude in the three tourism segments separately, Table 5 shows that, while the performance of the *city* segment is affected most by *Restaurant* workforce, the *mountain* destinations mostly rely on restaurants’ capital endowment. On the other hand, for maritime LMAs the most substantial input variable is labour coming from the *Accommodation* industry. Further insights on the heterogeneous behaviour of the input exploitation in these three destination typologies are observed for *Entertainment*. While only fixed assets have a significant effect on *sea* and *mountain* destinations, cities of arts and culture are exclusively influenced by labour coming from this sector. These results corroborate hypothesis H1, detecting substantial differences across tourism segments in cross-sectoral effects.

Besides internal inputs, the outputs of LMAs are also determined by neighbouring input factors. In particular, our results indicate that the Italian tourism sector is characterised by both diffusion and competition processes at the spatial level. Regarding the positive indirect effects, we find that neighbouring LMAs take advantage of positive externalities in line with the spatial agglomeration theory, mainly arising from capital investments in *Accommodation*, *Restaurant* and *Entertainment*. Concerning labour, we find a positive effect only for the *Transport* sector. These results support the idea that capital endowment in neighbouring areas is the most relevant input factor. Specifically, tourists can be attracted to neighbouring destinations due to preferences such as higher availability and range of activities, services, and facilities (Yang 2016). Considering competition processes, the first source of negative spillovers is associated with *Restaurant’*s labour. Indeed, negative effects on total tourism productive performance may depend on several characteristics of the restaurants’ labour market such as the high seasonality of the labour demand together with a scarcity of qualified workers in the sector (Smeral et al. 2004). Policymakers should design plans aimed at attracting workers into the sector, offering training programs and flexible conditions especially to young people. The idea is to motivate the entrance of new labour forces to strengthen the competitiveness of restaurants at the destination level.

The second source of negative spillovers concerns capital in the *Transport* sector. These results suggest that tourists prefer to visit easily accessible destinations that are connected through better infrastructure and passenger transport services (Ferri 2004), generating competitive pressures across neighbours (Masson and Petiot 2009). Possible suggestions dedicated to mitigating these competition effects across neighbours may concern specific investments for differentiating the tourism offer of neighbouring destinations. Indeed, providing different tourism products can help in limiting competition effects due to the uniqueness of the tourism amenities, services and attractions characterising a certain destination.

To conclude, even if policymakers are increasingly engaging in cooperative destination marketing to increase visitor flows and tourism expenditure in different destinations worldwide (Wing 1994a, b), several sustainable and developing programs should take into account the possible weaknesses in the production processes of sectors related to tourism such as restaurants and transport (Dwyer et al. 2003).

Finally, differentiating among tourism segments we find support in favour of our second research hypothesis H2: different destination typologies are characterised by different spatial processes. Focusing on the different contributions of labour and capital coming from neighbouring LMAs, we find that, while *city* destinations are affected more by the indirect effects of labour, *sea* and *mountain* outputs are influenced more by capital investment spillovers.

The main insight related to the *city* segment is found in the *Accommodation* sector. Of the three destination typologies, *city* is the only one affected by both classes of input spillovers from this sector, with positive significant spatial effects coming from labour and a negative impact from capital. This last feature suggests the need for a competitive renovation process of the facilities in the accommodation industry, aiming to renovate and modernise hotels and their portfolio of services (e.g. meeting rooms, spa, and entertainment) and to update their appeal.

Concerning *sea* and *mountain* destinations, we find that capital spillovers originating from all the tourism sectors, excluding *Transport*, appear to be positive and significant. Thus, local governments should design policies aimed at stimulating investments in the transport sector for both segments, with particular attention to *sea* destinations being the only ones characterised by negative spillover effects. A notable difference between *city* and the remaining segments concerns the indirect effects originating from the capital endowment of the *Creative&Arts* and *Entertainment* sectors. While the spillover effects associated with capital investments are statistically significant at a 5% significance level and positive in *sea* and *mountain* destinations, there is no empirical evidence in favour of this kind of spatial effects in *city* destinations. This result suggests that the presence of creative, cultural and entertainment facilities in coastal and landscape areas contributes to improving the tourism performance of neighbouring destinations. Thus, policymakers should invest in the development of cultural events and amenities, leisure activities and amusement spaces as complementary products to support the whole tourism supply in *sea* and *mountain* destinations.

### Robustness check

As robustness check, we first estimate the model in Eq. (2) considering the overall tourism industry without differentiating among the different sectors constituting it and then, we concentrate on the specific sectors identifying the tourism industry estimating single-sector models. Moreover, we also differentiate the analysis between tourism segments (the results referring to the single sectors are available from the authors under request).

The estimation results and the related short run and long run marginal effects are shown in Appendix respectively in Tables A7 and A8. The estimates of the overall model confirm that while positive capital spillovers significantly contribute to the productive performance of the tourism sector, negative spillover effects arise from labour force. Nevertheless, without differentiating among the input factors coming from the different sectors, it is not possible to detect the sources of such positive and negative spillovers. Distinguishing among tourism segments, we confirm that negative spatial effects related to capital and labour mainly occur in city and sea destinations respectively, while mountain destinations are characterised by positive capital spillovers.

Concentrating on the specific sectors constituting the tourism industry, we find that positive input spillovers overall characterise neighbouring destinations. However, in line with our previous results, we detect negative spatial effects associated with restaurants’ labour force and capital investments in the transport sector. However, differentiating among short run and long run effects, we find that while positive indirect effects mainly reinforce in time reaching a higher intensity, negative spillovers either decrease in magnitude or become not significant over a longer time horizon. Thus, policy makers should mainly concentrate on reinforcing cooperation and networking among neighbouring destinations in order to achieve large scale and long lasting results.

As a further robustness check, the model is estimated considering a different spatial weight matrix, to evaluate the robustness of the results. Table A9 in the Appendix shows the estimation results of the dynamic SDM with W built as first-order contiguity matrix. Findings are in line with the estimates obtained in Tables 3 and 4, showing only a small reduction in the spatial effects due to a slight decrease in the complexity of the network structure.

## Conclusion

In this study, we use aggregated data on the consolidated accounts of the different tourism sectors (*Accommodation*, *Restaurants*, *Creative&Arts*, *Entertainment*, and *Transports*) at the LMA level to investigate the productive performance of the Italian tourism industry in the period 2011–2020. The aim of the analysis is twofold: to evaluate cross-sectoral spillovers affecting the Italian tourism sector and detect spatial spillover effects influencing neighbouring destinations concentrating on three specific tourism segments (*city*, *sea*, and *mountain*) in order to investigate how these cross-sectoral and spatial phenomena vary considering specific Italian destination typologies. To achieve these goals, we estimate a dynamic spatial Durbin model in order to detect short run and long run input spillovers using a Cobb-Douglas function that allows us to evaluate how the input factors of the different sectors differently impact the overall tourism industry productivity. This is the first work modelling together cross-sectoral and spatial spillover effects in tourism, further combining two fundamental features for policy decisions: high spatial detail of analysis and the multipurpose nature of the tourism industry.

The results of the analysis confirm that the different sectors belonging to the same destination work together to build up a valuable tourism product. However, the role of the input factors in the productive process and the relationships constituting the structure of the network turn out to be strongly determined by the specific segment under analysis (H1), highlighting the necessity for policymakers to develop planning strategies for joint actions focusing on the specific features of the destinations.

Our spatial analysis shows that both productivity and input spillovers occur in the Italian tourism sector but, while global productivity spillovers positively influence all spatial units, either diffusion or competition input spillovers locally affect neighbouring destinations. In particular, there is a strong variability in the magnitudes and signs of input spillover effects across different tourism segments (H2). Then, even if we mostly observe improvement in the productive performance of neighbouring destinations due to agglomeration economies, negative input spillovers can also arise. Specifically, negative effects on the total value added of a destination in a certain territory are primarily due to restaurants’ labour and transports’ capital endowment, giving shape to local competition due to possible substitution effects (Ritchie and Crouch 2003).

From a managerial perspective, policymakers should be aware that local factors relating to specific tourism sectors exert an effective influence on the development of a given destination and the neighbouring areas. In particular, considering spillover effects coming from different tourism firms located in a certain destination, it is important to strengthen the cohesiveness of the network providing more opportunities for tourism organizations and sectors to learn from each other and raise awareness, for all the actors, of the importance of creating a collaborative environment to boost the competitiveness of the entire destination. At the same time, more attention should be paid to cross-destination spillovers, reinforcing pre-existing positive relationships and working on negative effects. Policymakers are increasingly engaging in cooperative destination marketing actions such as collective branding, joint promotions, and tour packages in collaboration with neighbouring destinations in order to take advantage of positive agglomeration economies (Dwyer et al. 2003). On the other hand, focusing on competition effects resulting from the multi-sectoral dimension of the tourism industry, it is fundamental for local institutions to bolster tourism destination competitiveness by investing in transport infrastructures and restaurant appeal, as they are the two factors that primarily suffer from competition spillovers. Programs or initiatives that may be developed by policymakers should concern the improvement of both the flexibility of the labour market and the training level of the employees of the restaurant sector, aiming to intensify the skills, aspirations and loyalty of employees to the sector. Focusing on the negative spillovers generated by the *Transport* sector, policymakers should pay attention to policies devoted to strengthening the accessibility of the areas and to bolstering the differentiation degrees of similar neighbouring destinations in terms of amenities, services, image and places of interest.

Finally, special attention should be paid to the creative and arts sector. Even if this sector never shows a direct impact on tourism destination value added, it contributes by boosting the performance of *sea* and *mountain* destinations through positive spatial spillover effects related to capital. Thus, fixed investments in this sector are beneficial to boosting the development of the whole local tourism industry.

Besides cross-sectoral and spatial spillover effects, further extensions of this work could also consider the distinction of cross-sectoral spillovers in intra- and inter-sectoral effects, extending the concept adopted by Badinger and Egger (2016) for OECD countries. Second, possible interaction effects across tourism segments can be investigated in view of the multi-purpose nature of the Italian tourism industry. Additionally, to obtain a more flexible functional form for the production function it would be interesting to extend the Cobb-Douglas function to a translog specification. This would allow not to impose any a priori restriction with respect to the internal returns to scale for the terms involving the input levels. However, the computational burden related to the estimation of a spatial translog function would strictly increase, leading to a weak parsimony and interpretability of the results (a complete spatial translog model would include also five squared terms of the inputs, 70 interaction terms between sectors by inputs, i.e. *lnL*_*its*_ *lnK*_*itz*_, *lnL*_*its*_ *lnL*_*itz*_, *lnK*_*its*_ *lnK*_*itz*_ for sectors s, z = {*Accommodation*, *Restaurant, Entertainment*, *Creative&Arts,Transport}*, and the associated spatial lags for these new sets of variables) Possible solutions will be investigated among the set of high-dimensionality econometric techniques recently developed. Finally, as kindly suggested by a reviewer, it could be the case that some areas are not exploiting their resources appropriately so that they are lagging behind their potential production level (i.e., productive inefficiency). Then, the analysis should be extended within a Stochastic Frontier framework, aiming at accounting for possible inefficient mechanisms in the tourism production process of destinations.

## Supplementary information

Appendix

## Footnotes

1 Retrievable at https://www.unwto.org/glossary-tourism-terms

2 Among others, in marketing see Morrison (2013) and Pike (2013); in destination branding and destination management organizations, Blain et al. (2005); in destination competitiveness Salinas Fernández et al. (2020); in tourism policymaking, Stevenson et al. (2008); in destination governance, Laws et al. (2011).

3 We have three completely missing spatial units for all five sectors (i.e. Casalnuovo Monterotaro in Apulia, Soriano Calabro in Calabria, and Perdasdefogu in Sardinia).
