# Supplementary material for: Spatial and cross-sectoral input spillover effects: the case of the Italian tourism industry
Source: J Product Anal. 2023 Feb 24;59(3):243–58. doi: 10.1007/s11123-023-00665-4 (PMC9951159; doi:10.1007/s11123-023-00665-4)

**Appendix**

Table A1. List of products reported in the Tourism Satellite Accounts

| Eurostat Classification |
| --- |
| A. Specific products |
| A.1 *Characteristic products* |
| 1. *Accommodation services* |
| 1.1 Hotels and similar |
| 1.2 Second homes—own account or free |
| 2. *Food and Beverage serving* |
| 3. *Passenger transports* |
| 3.1 Interurban railway transport |
| 3.2 Long distance road transport |
| 3.3 Water transport |
| 3.4 Air transport |
| 4. *Transport supporting services* |
| 5. *Transport equipment rental services* |
| 6. *Maintenance and repair of transportation equipment* |
| 7. *Travel agencies and other reservation services* |
| 8. *Cultural services* |
| 9. *Recreation and other entertainment services* |
| 10. *Miscellaneous / Other tourism services* |
| A.2 *Connected products* |
| B. Nonspecific products |
| B.2 *Other nonconsumption products* |

Table A2: Cross-correlation matrix between inputs and outputs by sectors

|  | lnQ_Acc_ | lnQ_Rest_ | lnQ_C&A_ | lnQ_Ent_ | lnQ_Tran_ | lnL_Acc._ | lnL_Rest._ | lnL_C&A_ | lnL_Ent._ | lnL_Tran._ | lnK_Acc._ | lnK_Rest._ | lnK_C&A_ | lnK_Ent._ | lnK_Tran._ |
| --- | --- | --- | --- | --- | --- | --- | --- | --- | --- | --- | --- | --- | --- | --- | --- |
| lnQ_Acc._ | 1.00 |  |  |  |  |  |  |  |  |  |  |  |  |  |  |
| lnQ_Rest._ | 0.62 | 1.00 |  |  |  |  |  |  |  |  |  |  |  |  |  |
| lnQ_C&A_ | 0.37 | 0.55 | 1.00 |  |  |  |  |  |  |  |  |  |  |  |  |
| lnQ_Ent._ | 0.59 | 0.70 | 0.54 | 1.00 |  |  |  |  |  |  |  |  |  |  |  |
| lnQ_Tran._ | 0.53 | 0.53 | 0.44 | 0.52 | 1.00 |  |  |  |  |  |  |  |  |  |  |
| lnL_Acc._ | 0.94 | 0.63 | 0.42 | 0.61 | 0.55 | 1.00 |  |  |  |  |  |  |  |  |  |
| lnL_Rest._ | 0.61 | 0.96 | 0.61 | 0.73 | 0.55 | 0.64 | 1.00 |  |  |  |  |  |  |  |  |
| lnL_C&A_ | 0.35 | 0.51 | 0.88 | 0.49 | 0.42 | 0.40 | 0.57 | 1.00 |  |  |  |  |  |  |  |
| lnL_Ent._ | 0.58 | 0.69 | 0.59 | 0.90 | 0.51 | 0.61 | 0.74 | 0.55 | 1.00 |  |  |  |  |  |  |
| lnL_Tran._ | 0.52 | 0.56 | 0.51 | 0.54 | 0.95 | 0.55 | 0.59 | 0.50 | 0.55 | 1.00 |  |  |  |  |  |
| lnK_Acc._ | 0.93 | 0.59 | 0.34 | 0.56 | 0.50 | 0.87 | 0.58 | 0.33 | 0.54 | 0.49 | 1.00 |  |  |  |  |
| lnK_Rest._ | 0.61 | 0.93 | 0.50 | 0.66 | 0.51 | 0.61 | 0.89 | 0.44 | 0.64 | 0.54 | 0.58 | 1.00 |  |  |  |
| lnK_C&A_ | 0.38 | 0.55 | 0.89 | 0.53 | 0.44 | 0.43 | 0.61 | 0.81 | 0.59 | 0.51 | 0.36 | 0.50 | 1.00 |  |  |
| lnK_Ent._ | 0.58 | 0.69 | 0.50 | 0.89 | 0.49 | 0.59 | 0.71 | 0.45 | 0.81 | 0.50 | 0.55 | 0.65 | 0.50 | 1.00 |  |
| lnK_Tran._ | 0.52 | 0.51 | 0.42 | 0.50 | 0.96 | 0.54 | 0.52 | 0.40 | 0.49 | 0.92 | 0.50 | 0.49 | 0.42 | 0.47 | 1.00 |

Table A3: Moran test on value added (*lnQ*)

| Year | Moras'I | E(I) | Sd(I) | Z | P-value |
| --- | --- | --- | --- | --- | --- |
| 2011 | 0.211 | -0.002 | 0.016 | 13.683 | 0.000 |
| 2012 | 0.215 | -0.002 | 0.016 | 13.914 | 0.000 |
| 2013 | 0.207 | -0.002 | 0.016 | 13.386 | 0.000 |
| 2014 | 0.198 | -0.002 | 0.016 | 12.801 | 0.000 |
| 2015 | 0.204 | -0.002 | 0.016 | 13.204 | 0.000 |
| 2016 | 0.195 | -0.002 | 0.016 | 12.593 | 0.000 |
| 2017 | 0.202 | -0.002 | 0.016 | 13.072 | 0.000 |
| 2018 | 0.204 | -0.002 | 0.016 | 13.164 | 0.000 |
| 2019 | 0.207 | -0.002 | 0.016 | 13.369 | 0.000 |
| 2020 | 0.278 | -0.002 | 0.016 | 17.937 | 0.000 |

Table A4: Moran test on input factors: labour (*lnL*) and capital (*lnK*) by sectors

|  | lnL | | | lnK | | |
| --- | --- | --- | --- | --- | --- | --- |
| Year | Moran’s I | Z | P-value | Moran’s I | Z | P-value |
| Accommodation |  |  |  |  |  |  |
| 2011 | 0.158 | 10.196 | 0.000 | 0.147 | 9.504 | 0.000 |
| 2012 | 0.168 | 10.848 | 0.000 | 0.149 | 9.705 | 0.000 |
| 2013 | 0.159 | 10.334 | 0.000 | 0.153 | 9.868 | 0.000 |
| 2014 | 0.154 | 9.953 | 0.000 | 0.152 | 9.870 | 0.000 |
| 2015 | 0.166 | 10.759 | 0.000 | 0.169 | 10.997 | 0.000 |
| 2016 | 0.163 | 10.532 | 0.000 | 0.173 | 11.206 | 0.000 |
| 2017 | 0.168 | 10.862 | 0.000 | 0.180 | 11.673 | 0.000 |
| 2018 | 0.168 | 10.849 | 0.000 | 0.185 | 11.960 | 0.000 |
| 2019 | 0.181 | 11.706 | 0.000 | 0.190 | 12.296 | 0.000 |
| 2020 | 0.210 | 13.556 | 0.000 | 0.233 | 15.008 | 0.000 |
| Restaurants |  |  |  |  |  |  |
| 2011 | 0.199 | 12.904 | 0.000 | 0.160 | 10.373 | 0.000 |
| 2012 | 0.190 | 12.301 | 0.000 | 0.167 | 10.816 | 0.000 |
| 2013 | 0.191 | 12.324 | 0.000 | 0.168 | 10.864 | 0.000 |
| 2014 | 0.189 | 12.259 | 0.000 | 0.160 | 10.374 | 0.000 |
| 2015 | 0.184 | 11.904 | 0.000 | 0.159 | 10.319 | 0.000 |
| 2016 | 0.196 | 12.686 | 0.000 | 0.155 | 10.049 | 0.000 |
| 2017 | 0.211 | 13.601 | 0.000 | 0.182 | 11.767 | 0.000 |
| 2018 | 0.196 | 12.671 | 0.000 | 0.184 | 11.929 | 0.000 |
| 2019 | 0.202 | 13.058 | 0.000 | 0.188 | 12.146 | 0.000 |
| 2020 | 0.256 | 16.506 | 0.000 | 0.225 | 14.505 | 0.000 |
| Entertainment |  |  |  |  |  |  |
| 2011 | 0.048 | 3.203 | 0.000 | 0.078 | 5.095 | 0.000 |
| 2012 | 0.048 | 3.166 | 0.000 | 0.083 | 5.425 | 0.000 |
| 2013 | 0.061 | 4.022 | 0.000 | 0.088 | 5.738 | 0.000 |
| 2014 | 0.053 | 3.521 | 0.000 | 0.081 | 5.318 | 0.000 |
| 2015 | 0.040 | 2.673 | 0.000 | 0.079 | 5.182 | 0.000 |
| 2016 | 0.058 | 3.805 | 0.000 | 0.089 | 5.838 | 0.000 |
| 2017 | 0.067 | 4.368 | 0.000 | 0.097 | 6.336 | 0.000 |
| 2018 | 0.046 | 3.058 | 0.000 | 0.102 | 6.612 | 0.000 |
| 2019 | 0.046 | 3.041 | 0.000 | 0.095 | 6.214 | 0.000 |
| 2020 | 0.051 | 3.369 | 0.000 | 0.079 | 5.147 | 0.000 |
| Creative&Arts |  |  |  |  |  |  |
| 2011 | 0.143 | 9.229 | 0.000 | 0.186 | 12.016 | 0.000 |
| 2012 | 0.148 | 9.561 | 0.000 | 0.203 | 13.069 | 0.000 |
| 2013 | 0.154 | 9.975 | 0.000 | 0.196 | 12.632 | 0.000 |
| 2014 | 0.171 | 11.024 | 0.000 | 0.199 | 12.817 | 0.000 |
| 2015 | 0.142 | 9.215 | 0.000 | 0.192 | 12.404 | 0.000 |
| 2016 | 0.165 | 10.659 | 0.000 | 0.203 | 13.119 | 0.000 |
| 2017 | 0.166 | 10.717 | 0.000 | 0.206 | 13.315 | 0.000 |
| 2018 | 0.167 | 10.771 | 0.000 | 0.232 | 14.968 | 0.000 |
| 2019 | 0.165 | 10.649 | 0.000 | 0.239 | 15.400 | 0.000 |
| 2020 | 0.201 | 12.972 | 0.000 | 0.277 | 17.841 | 0.000 |
| Transports |  |  |  |  |  |  |
| 2011 | 0.078 | 5.105 | 0.000 | 0.141 | 9.121 | 0.000 |
| 2012 | 0.083 | 5.429 | 0.000 | 0.135 | 8.736 | 0.000 |
| 2013 | 0.089 | 5.802 | 0.000 | 0.134 | 8.656 | 0.000 |
| 2014 | 0.085 | 5.517 | 0.000 | 0.145 | 9.407 | 0.000 |
| 2015 | 0.095 | 6.202 | 0.000 | 0.141 | 9.106 | 0.000 |
| 2016 | 0.096 | 6.221 | 0.000 | 0.144 | 9.348 | 0.000 |
| 2017 | 0.096 | 6.269 | 0.000 | 0.137 | 8.877 | 0.000 |
| 2018 | 0.093 | 6.026 | 0.000 | 0.134 | 8.664 | 0.000 |
| 2019 | 0.088 | 5.741 | 0.000 | 0.132 | 8.548 | 0.000 |
| 2020 | 0.127 | 8.207 | 0.000 | 0.185 | 11.941 | 0.000 |

Table A5: VIF multicollinearity tests after least square estimation

| Variable | VIF |
| --- | --- |
| lnL_Accommodation_ | 4.82 |
| lnL_Restaurant_ | 7.19 |
| lnL_Creative&Arts_ | 3.06 |
| lnL_Entertainment_ | 3.86 |
| lnL_Transport_ | 7.71 |
|  |  |
| lnK_Accommodation_ | 4.22 |
| lnK_Restaurant_ | 5.14 |
| lnK_Creative&Arts_ | 3.26 |
| lnK_Entertainment_ | 3.27 |
| lnK_Transport_ | 6.78 |

Table A6: Chow test: chi-square statistics

|  | *H_0_:*  *Coeff._City_= Coeff._Sea_* | *H_0_:*  *Coeff._City_= Coeff._Mountain_* | *H_0_:*  *Coeff._Sea_= Coeff._Mountain_* |
| --- | --- | --- | --- |
| lnL_Accommodation_ | 15.93*** | 2.69* | 10.12*** |
| lnL_Restaurant_ | 0.76 | 0.43 | 3.82** |
| lnL_Creative&Arts_ | 0.12 | 0.04 | 0.30 |
| lnL_Entertainment_ | 6.11** | 2.52 | 1.70 |
| lnL_Transport_ | 1.72 | 2.30 | 0.07 |
| lnK_Accommodation_ | 1.96 | 5.12** | 0.53 |
| lnK_Restaurant_ | 2.27 | 1.53 | 17.04*** |
| lnK_Creative&Arts_ | 0.01 | 0.02 | 0.00 |
| lnK_Entertainment_ | 0.88 | 3.17* | 0.91 |
| lnK_Transport_ | 1.37 | 0.01 | 2.77* |
| WlnL_Accommodation_ | 0.41 | 3.07* | 7.83*** |
| WlnL_Restaurant_ | 4.66** | 7.60*** | 0.35 |
| WlnL_Creative&Arts_ | 0.50 | 1.79 | 0.31 |
| WlnL_Entertainment_ | 8.18*** | 0.34 | 14.61*** |
| WlnL_Transport_ | 5.59** | 5.42** | 0.00 |
| WlnK_Accommodation_ | 17.89*** | 7.71*** | 6.65*** |
| WlnK_Restaurant_ | 0.00 | 0.02 | 0.02 |
| WlnK_Creative&Arts_ | 0.05 | 0.12 | 0.55 |
| WlnK_Entertainment_ | 1.28 | 0.14 | 0.95 |
| WlnK_Transport_ | 0.89 | 3.24* | 1.49 |

***:p-value<=0.01;**:p-value<=0.05;*:p-value<=0.1

Table A7. Robustness Check: Estimated Coefficients

|  | All sectors | | | | Single sector | | | | |
| --- | --- | --- | --- | --- | --- | --- | --- | --- | --- |
|  | Overall | City | Sea | Mount. | Accomm. | Rest. | Creat. | Entert. | Transp. |
| ${lnQ}_{t-1}$ | 0.27*** | 0.09*** | 0.11*** | 0.26*** | 0.23*** | 0.18*** | 0.29*** | 0.27*** | 0.16*** |
|  | (0.01) | (0.03) | (0.03) | (0.02) | (0.01) | (0.01) | (0.01) | (0.01) | (0.01) |
| ${WlnQ}_{t}$ | 0.42*** | 0.43*** | 0.24*** | 0.20*** | 0.32*** | 0.40*** | 0.03 | 0.21*** | 0.27*** |
|  | (0.03) | (0.03) | (0.03) | (0.01) | (0.03) | (0.03) | (0.03) | (0.03) | (0.03) |
| lnL | 0.58*** | 0.57*** | 0.80*** | 0.65*** | 0.62*** | 0.61*** | 0.49*** | 0.44*** | 0.80*** |
|  | (0.02) | (0.03) | (0.03) | (0.03) | (0.02) | (0.02) | (0.02) | (0.02) | (0.02) |
| lnK | 0.37*** | 0.23*** | 0.22*** | 0.45*** | 0.35*** | 0.40*** | 0.42*** | 0.37*** | 0.39*** |
|  | (0.01) | (0.02) | (0.02) | (0.02) | (0.01) | (0.01) | (0.01) | (0.01) | (0.01) |
| WlnL | -0.25*** | -0.04 | -0.16*** | 0.02 | 0.01 | -0.39*** | 0.14** | 0.04 | 0.24*** |
|  | (0.04) | (0.05) | (0.05) | (0.06) | (0.05) | (0.04) | (0.06) | (0.05) | (0.07) |
| WlnK | 0.14*** | -0.16*** | 0.13*** | 0.07* | 0.02 | 0.04 | 0.07* | 0.10*** | -0.18*** |
|  | (0.04) | (0.05) | (0.04) | (0.04) | (0.03) | (0.03) | (0.04) | (0.03) | (0.04) |

Standard errors in brackets

***:p-value<=0.01;**:p-value<=0.05;*:p-value<=0.1

Table 8. Robustness Check: Marginal Effects

|  |  |  | All sectors | | | | Single sector | | | | |
| --- | --- | --- | --- | --- | --- | --- | --- | --- | --- | --- | --- |
|  |  |  | Overall | City | Sea | Mount | Accom | Rest. | Creat. | Entert | Transp |
| Short Run | *lnL* | Dir. | 0.58^***^ | 0.59^***^ | 0.80^***^ | 0.65^***^ | 0.62^***^ | 0.60^***^ | 0.49^***^ | 0.45^***^ | 0.81^***^ |
|  |  | Ind. | -0.02 | 0.31^***^ | 0.04 | 0.18^***^ | 0.30^***^ | -0.23^***^ | 0.15^***^ | 0.16^***^ | 0.61^***^ |
|  | *lnK* | Dir. | 0.38^***^ | 0.23^***^ | 0.23^***^ | 0.43^***^ | 0.35^***^ | 0.41^***^ | 0.42^***^ | 0.37^***^ | 0.39^***^ |
|  |  | Ind. | 0.50^***^ | -0.08 | 0.22^***^ | 0.19^***^ | 0.19^***^ | 0.33^***^ | 0.09^***^ | 0.21^***^ | -0.10^**^ |
| Long Run | *lnL* | Dir. | 0.80^***^ | 0.66^***^ | 0.89^***^ | 0.88^***^ | 0.81^***^ | 0.73^***^ | 0.69^***^ | 0.62^***^ | 0.97^***^ |
|  |  | Ind. | 0.25^***^ | 0.41^***^ | 0.08 | 0.34^***^ | 0.58^***^ | -0.21^***^ | 0.23^***^ | 0.30^***^ | 0.85^***^ |
|  | *lnK* | Dir. | 0.54^***^ | 0.26^***^ | 0.27^***^ | 0.62^***^ | 0.46^***^ | 0.50^***^ | 0.59^***^ | 0.51^***^ | 0.46^***^ |
|  |  | Ind. | 1.12^***^ | -0.08 | 0.26^***^ | 0.34^***^ | 0.35^***^ | 0.54^***^ | 0.13** | 0.37^***^ | -0.10 |

***:p-value<=0.01;**:p-value<=0.05;*:p-value<=0.1

Table A9. Robustness Check: Sensitivity to the Choice of W

|  | *Overall* | | *City* | | *Sea* | | *Mountain* | |
| --- | --- | --- | --- | --- | --- | --- | --- | --- |
|  | *Coeff.* | *SD* | *Coeff.* | *SD* | *Coeff.* | *SD* | *Coeff.* | *SD* |
| lnQ_t-1_ | 0.43*** | 0.01 | 0.15*** | 0.03 | 0.24*** | 0.03 | 0.48*** | 0.02 |
| WlnQ_t_ | 0.15*** | 0.02 | 0.20*** | 0.03 | 0.14*** | 0.02 | 0.11*** | 0.02 |
| lnL_Accommodation_ | 0.12*** | 0.01 | 0.08*** | 0.02 | 0.27*** | 0.02 | 0.11*** | 0.02 |
| lnL_Restaurant_ | 0.11*** | 0.02 | 0.21*** | 0.03 | 0.13*** | 0.02 | 0.15*** | 0.03 |
| lnL_Creative&Arts_ | 0.02 | 0.01 | 0.01 | 0.01 | 0.02 | 0.02 | 0.02 | 0.02 |
| lnL_Entertainment_ | 0.02* | 0.01 | 0.09*** | 0.01 | 0.01 | 0.02 | 0.03 | 0.02 |
| lnL_Transport_ | 0.13*** | 0.01 | 0.16*** | 0.02 | 0.15*** | 0.02 | 0.11*** | 0.03 |
| lnK_Accommodation_ | 0.09*** | 0.01 | 0.04*** | 0.01 | 0.09*** | 0.01 | 0.10*** | 0.01 |
| lnK_Restaurant_ | 0.12*** | 0.01 | 0.10*** | 0.02 | 0.06*** | 0.01 | 0.14*** | 0.01 |
| lnK_Creative&Arts_ | 0.01 | 0.01 | 0.02 | 0.01 | 0.01 | 0.01 | 0.01 | 0.01 |
| lnK_Entertainment_ | 0.05*** | 0.01 | 0.02 | 0.01 | 0.05*** | 0.01 | 0.06*** | 0.01 |
| lnK_Transport_ | 0.02** | 0.01 | 0.04*** | 0.01 | 0.01 | 0.01 | 0.03** | 0.01 |
| WlnL_Accommodation_ | 0.04 | 0.03 | 0.11*** | 0.03 | -0.04 | 0.02 | -0.01 | 0.04 |
| WlnL_Restaurant_ | -0.12*** | 0.03 | -0.08** | 0.04 | -0.04 | 0.03 | -0.07* | 0.04 |
| WlnL_Creative&Arts_ | 0.04* | 0.02 | 0.01 | 0.02 | 0.02 | 0.02 | -0.01 | 0.04 |
| WlnL_Entertainment_ | -0.01 | 0.02 | -0.01 | 0.02 | -0.01 | 0.02 | 0.01 | 0.03 |
| WlnL_Transport_ | 0.16*** | 0.03 | 0.13*** | 0.03 | 0.01 | 0.02 | 0.15*** | 0.05 |
| WlnK_Accommodation_ | 0.09*** | 0.02 | -0.10*** | 0.03 | 0.10*** | 0.02 | 0.05*** | 0.02 |
| WlnK_Restaurant_ | 0.08*** | 0.02 | -0.02 | 0.03 | 0.05** | 0.02 | 0.07*** | 0.02 |
| WlnK_Creative&Arts_ | 0.04** | 0.01 | 0.02 | 0.02 | 0.01 | 0.01 | 0.04** | 0.02 |
| WlnK_Entertainment_ | 0.06*** | 0.01 | 0.02 | 0.02 | 0.03** | 0.01 | 0.06*** | 0.02 |
| WlnK_Transport_ | -0.04** | 0.02 | -0.02 | 0.02 | -0.02 | 0.01 | -0.03 | 0.03 |

***: p-value<0.01; **: p-value<0.05; *: p-value<0.10.

Figure A1: scatterplots between total value added and inputs by sectors

| 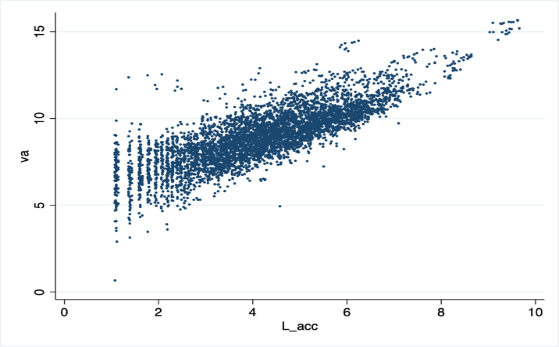 | 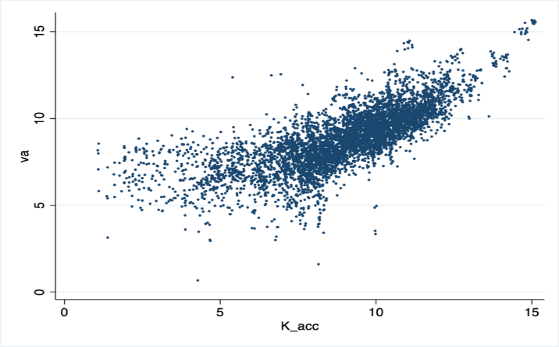 |
| --- | --- |
| 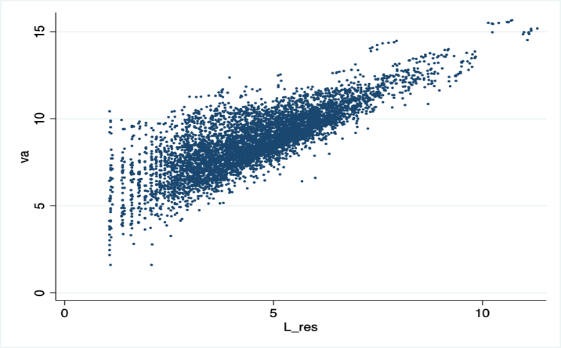 | 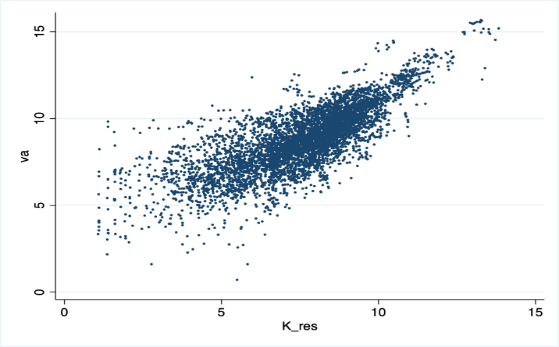 |
| 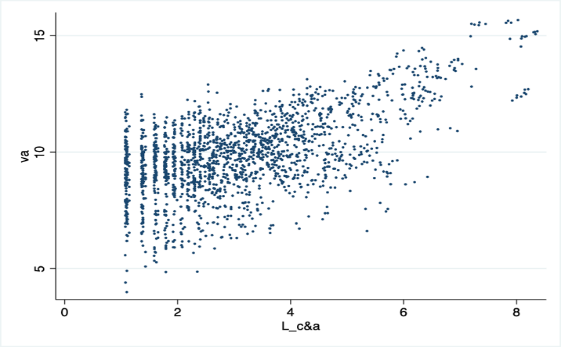 | 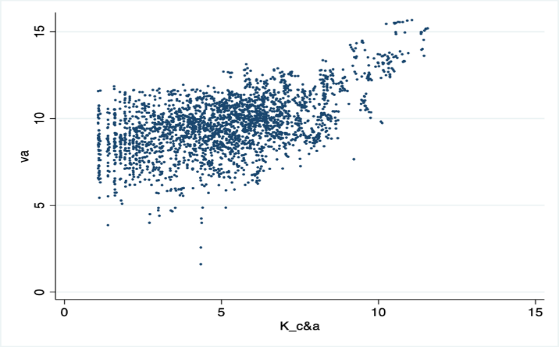 |
| 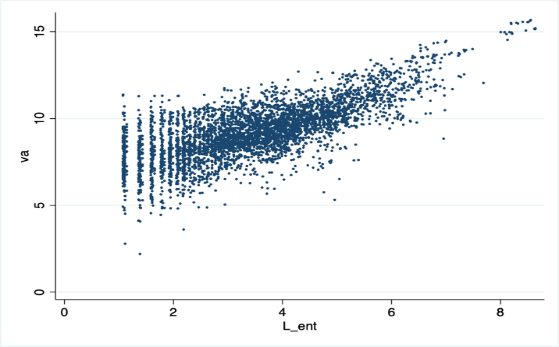 | 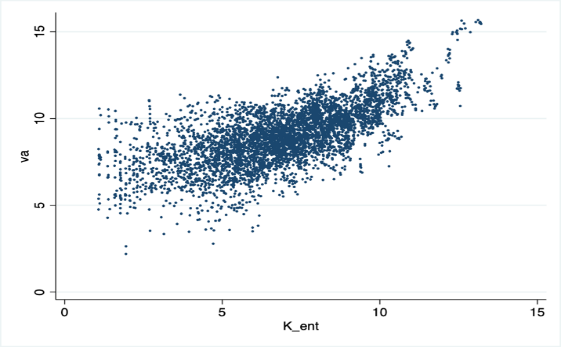 |
| 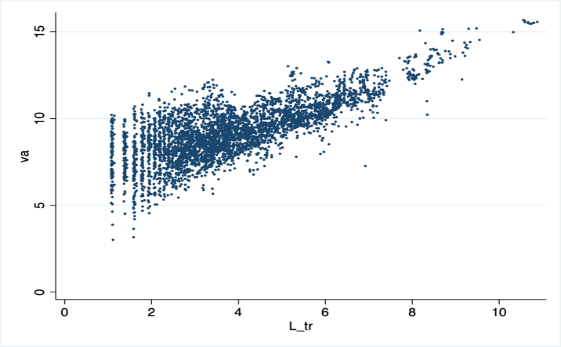 | 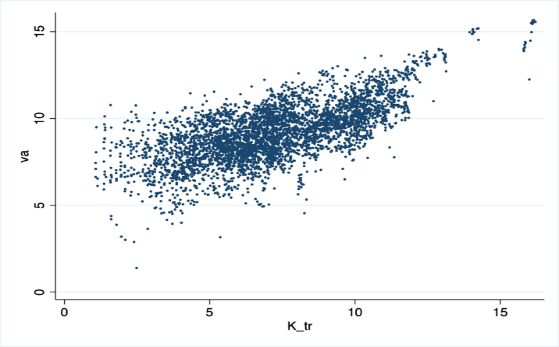 |

Figure A2. Italian Regions

Figure A3. LISA Significance Cluster Map: Value Added 2019


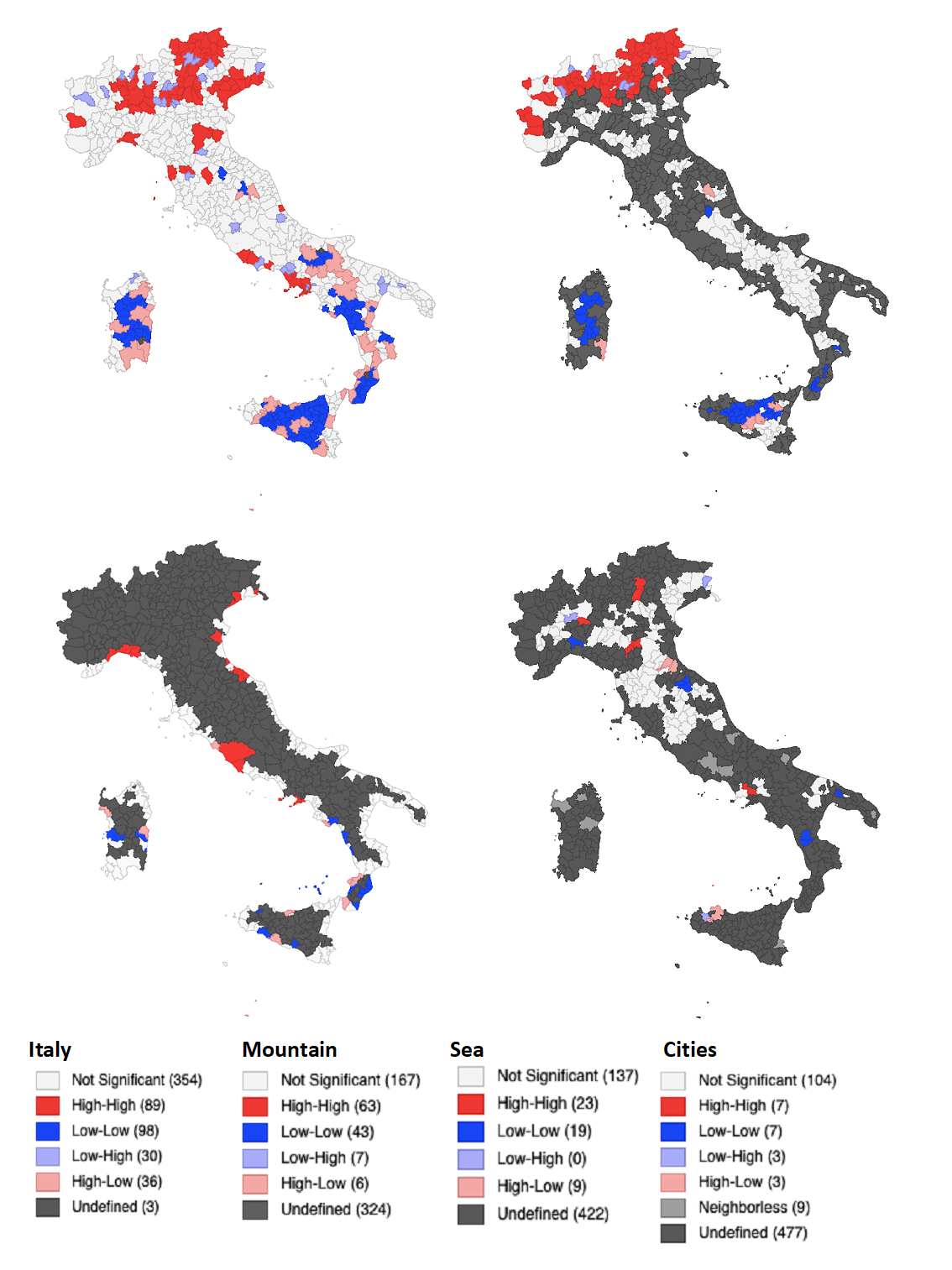


Figure A4. Individual fixed effects: percentile map


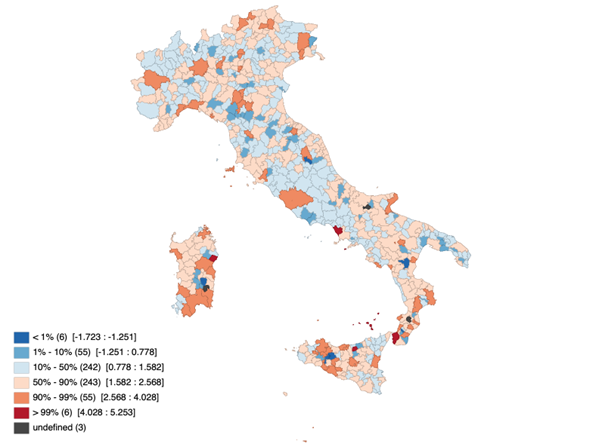

Supplement: Supplementary file 1 — Appendix [file 11123_2023_665_MOESM1_ESM.docx]
